# Editorial: Patriarchy and Populism During the COVID-19 Pandemic

**DOI:** 10.3389/fsoc.2021.722393

**Published:** 2021-08-06

**Authors:** Carol C. Gould

**Affiliations:** Department of Philosophy, Hunter College, and PhD Programs in Political Science and Philosophy, The Graduate Center, City University of New York, New York, NY, United States

**Keywords:** populism, gender, patriarchy, democracy, nationalism, authoritarianism, intersectionality, social movements

## Motivation, Aims, and Methods

Over the last decade, we have been confronted around the world not only with an authoritarian populist movement but also with an increasingly white supremacist one. Likewise, authoritarian rulers have found support from right-wing populist groupings, which tend to exhibit nationalist, nativist, and xenophobic ideologies. A striking feature of most of these movements is the predominance of men among their membership and leadership, along with a noteworthy tendency to espouse male dominant or sexist standpoints and to advocate a return to explicitly patriarchal practices in family and social life (Spierings et al., 2015; Moghadam and Kaftan, 2019; Löffler et al., 2020; Sauer, 2020).

The idea for this special issue on populism and patriarchy originated at the 2019 annual convention of the American Political Science Association, when its co-editors separately observed that nearly all the discussions of populism failed to mention the role or the relevance of gender. We heard no analysis of the linkages between right-wing populism and patriarchal practice, or of the predominance of male chauvinism or sexism in the perspectives and processes of social movements. The editors of this Research Topic decided to organize a roundtable at the 2020 APSA annual meeting that would focus on these issues. We invited a group of accomplished political theorists to participate and to bring their own distinctive analyses to bear.

All the talks and resulting papers were written at the height of the virulent COVID-19 pandemic, which has been raging across the globe and causing enormous hardship and millions of deaths. In the United States and elsewhere, we witnessed widespread tendencies to disregard the recommendations of public health experts, encouraged by the same right-wing populist groups. Millions refused to wear masks, to limit public gatherings, or otherwise act to protect others, as was (normatively) required. Literally toxic notions of masculinity were invoked and practiced, raising again the crucial issue of the (in this case deadly) intersection of populism with patriarchy. At the same time, increasingly segmented social media enabled the expression and perpetuation of conspiracy theories and white male supremacist organizing, with pernicious and often violent consequences.

The papers collected in this special issue explore this nexus of issues in ways that go beyond the existing (and by now considerable) literature on the confluence of populism with patriarchal ideas, sentiments, and modes of action (among them, Dietze and Roth, 2020; Graff et al., 2019). What this collection specifically aims to do is to use the distinctive methods and expertise of political and social theory to illuminate some of the key issues raised by these troubling contemporary developments, and to clarify some important questions for future inquiry. This collection includes analytical, normative, and empirical approaches.

### Framing the Research Topic: Understanding Gender and Authoritarian Populism in Terms of Intersecting Axes of Oppression

By way of introduction, consider the significance of the fact that the majority of adherents to right-wing populist movements are cis men, although it is also worth noting the avid participation of some women (which itself demands explanation). Movement members, regardless of gender, tend to blame feminists and other gender egalitarians, along with Black people, Muslims, migrants, and other minorities, for the various problems they face. Clearly, a motivating factor seems to be a reaction against various feminist achievements, especially women’s increasing participation and equality at work (Sauer, 2020, 29–30). Both membership and leadership tend to advocate a return to more traditional forms of family life, with male dominance in those contexts, as breadwinners and heads of family, along with the naturalization of sexual and gender difference.

Yet, the concurrence of these patriarchal attitudes and practices with white supremacist ones suggests the utility of an intersectional approach. I would propose that we consider intersectionality here, not only in its conventional application to the targets of oppression, but also in understanding the agents and systems of oppression themselves. Crucially, the axes of oppression intersect within oppressor groups too, bringing together the affirmation of gender inequality, racist ideology, and anti-democratic/authoritarian attitudes in politics. Some of these views are religiously based, most commonly claiming sources in evangelical Christianity, but sometimes also finding them in Judaism, Hinduism, or Islam. The religious dimension, however, is not a necessary condition of the authoritarian populism under consideration here.

The issue of class composition is a bit more complex, since right-wing populist movements tend to attract members of both the middle and working classes, a large number of them non-college educated workers, but also a significant segment of middle managers and small business owners. Some members of economic elites are involved, as well, and leaders tend to come from that socio-economic stratum.

How, then, can we understand the rabidly anti-feminist agenda of these social movements and political figures? Why have these and other forms of sexism and male chauvinism been systematically linked to authoritarian populism, and how can feminist theory contribute to the cultivation of an alternative egalitarian and even emancipatory framework?

We have here a range of right-populist phenomena, which have sometimes been inaccurately lumped together with *popular* social movements on the left, in Latin America and elsewhere. The term *populism* is by now widely recognized as a fundamentally ambiguous notion. Some of the articles in this collection focus on its diverse meanings and attempt to give us a more substantive understanding of the concept. The version that is most problematic from a feminist standpoint could be called authoritarian populism, which in some countries has now turned into authoritarianism *simpliciter*. The populist dimension signifies the existence of social and political movements that embrace charismatic leaders with authoritarian tendencies, who claim to speak in the name of the people (Morelock, 2018, xvi). In fact, such movements can also be analyzed as counter-movements to the perceived successes of the movements for racial and gender equality.

Each of the articles in this collection advances their own take on populism, but most theorists agree that we need to distinguish between left and right forms. While all types of populism appeal to a notion of a people standing up to “elites,” right-wing renditions tend to posit a third group which is somehow responsible for the problems the “real” or “authentic” people face, blaming these others for their situation (Judis, 2016, 14–15). In the cases examined here, feminists are prominent in that third group, as are Blacks and other non-white people, as well as non-citizens and non-Christians, especially Muslims and Jews, who deviate from the ethnos or racialized people of choice.

Not everybody can be part of “the people” in such right-wing populist conceptions. This is distinct from those of popular movements on the left. These, at least, aspire to be horizontal and fully democratic in their functioning, and valorize consensus decision-making and a culture of consent, even if they do not always exemplify these in practice (Gould-Wartofsky, 2015). By contrast, in populist movements on the right, neither equality, nor democracy, nor solidarity are understood in terms of any benefits they could bring, but only as diminishing their members’ standing.

The question remains as to why there is such a heavily gendered aspect in these right-wing movements and what accounts for their role in the recent resurgence of patriarchal politics. Why are anti-feminism and anti-gender-pluralism so closely correlated with this phenomenon of authoritarian populism?

The full answer is elusive, and the situation is complex. One source of right-populism is the spread of neoliberal capitalism over the last several decades (Brown, 2019), with its tendencies to outsource employment and to intensify existing inequalities in wealth, income, and ownership. These have affected not only the traditional working class, but the middle classes as well, including small business owners. While those impacted have grievances which are legitimate, right-wing populism tends to channel these away from the sources of their grievances, and towards populations that are not in fact responsible for their problems (and who themselves also suffer from them).

Indeed, oddly, and indeed paradoxically, right-wing populist groups have often supported leaders—like Donald Trump—who are themselves members of this elite and who have in reality contributed to the problems that the populist group members face. They may find common cause in shared notions of whiteness and of male-dominant masculinities. In the United States case, for example, they not only failed to hold former President Trump responsible for the sexual predations of which he has been accused, but even embraced the model of masculinity he claimed to represent.

Instead of working to ameliorate structural inequalities or address the pernicious effects of neoliberal capitalism, these movements function to reinforce these structures and reproduce their power. As a result, they fail to address the real causes of people’s lack of power and their accompanying feelings of powerlessness. From this standpoint, expressing grievances and lashing out against groups of “others” serves as a distraction from the resolution of these grievances, and may in fact serve the interest of the elites. Of course, we need not attribute malign intent to the latter to understand such a diversionary outcome.

Beyond the inequalities of wealth and income, then, individuals rightly perceive an imbalance of power, both at work and in social life beyond it. While members of populist groups may lack power at work, they do not see the way this characterizes working people as a class. Moreover, from the standpoint of feminist theory, we can say that members mistake the desirable forms of power as consisting in “power over” others rather than “power with” others (Allen, 1998; Gould, 2014). This view of power as control or domination over others is itself facilitated by the operations of capitalism, with its inherently competitive modes of interaction and its hierarchical modes of organization. But these misreadings of power may also derive from upbringings within parental contexts that also tend to identify such “power over” others as the only type that counts, or the sole criterion of success. This observation suggests the relevance of the “authoritarian personality” (Theodor et al., 1950; Gordon, 2018), which works against the sort of democratic personality needed to support more cooperative modes of governance (Gould, 1988, 282–306).

In addition to the intersection of racism, sexism, nationalism, and capitalism, we need to ask again what benefits the participants derive from the resurrection of patriarchal populism. The answer is somewhat simple and direct—they believe they will have more and better opportunities if they suppress the prospects of others, e.g., that they will do better if women do not get jobs, and/or if the male members are able to exert customary forms of chauvinism and superiority in their sexual relations as well. Here, again, superiority is taken as *power over* in a structurally competitive framework. Yet, in this way, populists are also buying into and perpetuating the standards implicit in capitalism and neoliberalism that have in fact denied these people opportunities, and where some can gain only if others lose. It is clear too that the form of feminism targeted by patriarchal populists is a simplistic version, centering exclusively on equal opportunity as its core value, in which the goal is only for women to become managers and to be paid equally. However important these sorts of achievements may be, they do not yet engage the claims of more political forms of feminism, which argue for the need to participate in projects of fundamental social transformation as a condition for full and genuine equality.

#### Feminist Ethics and the Possibility of Social and Political Transformation

In contrast to the emphasis among right-wing populists on the loss of “perks” and the restoration of their power over others, feminists and other progressively minded groups see real power as instantiated in *power with* others. They argue that new modes of cooperative care and shared responsibility, when implemented structurally and not only interpersonally, can be beneficial for everyone and allow all to develop themselves more fully. Clearly, deeper forms of democratic participation would be essential in this perspective, with an extension of democratic modes of decision-making beyond politics to work, as in worker control and workplace democracy. Indeed, it can be expected that less hierarchy at work could help to address feelings of powerlessness, too, though these changes would not be sufficient in that regard (Gould, 2019).

Although it is difficult to say what could change the present dynamics of gender inequality, gender roles, and their reproduction in society, it would be important to address the tendency of many men to identify with aggressive or even violent male role models. Clearly, the caring and collaborative dimensions of experience would have to become more appealing to people, and more highly valued. Likewise, the public domains of life—whether economic, social, or political—would benefit from specifically cooperative forms of organization, along with democratic management, accountability, and worker autonomy. Fuller provision by political communities of the necessary conditions for the development of talents and the cultivation of relations would be required. The sort of equality called for here is not to be understood as sameness, but as equality in the basic conditions for differentiated individuals. In this reading, the freedom for each would be seen as a condition for freedom for all. With growing recognition of interdependence and the benefits of cooperation, the hope is that people would come to appreciate the ways in which their own freedom can be enhanced, rather than minimized, by the free development of others.

Feminist ethics has long argued for placing a greater emphasis on responsibility, rather than solely focusing on individual rights (Held, 2006; Robinson, 2006). From this perspective, the social and political organization needed to fulfill basic rights can be understood in terms of ongoing shared responsibilities rather than as particular duties that can be discharged once and for all. More relational notions of autonomy or, as I prefer, self-development, also draw on feminist ontologies and epistemologies (Gould, 1988; Gould, 2014). While movements against gender, racial, sexual, and religious oppression each have their own validity and emphases, they can helpfully see themselves as intersecting with and drawing on social movements for economic equality, and as enacting norms of inclusivity and democracy. In the context of politics, such inclusivity requires that all those residing within a political community (including the undocumented) be regarded as equal members. Effecting the necessary changes will require not only real political power but also, crucially, social movements that construct relations of solidarity with each other across borders. The goal is not only formal equality but also the provision of the social, material, and political conditions that all need for their self-development, replacing the present system in which only some have access to these conditions and make use of them at the expense of others.

#### Highlighting the Diverse Perspectives and Overlapping Concerns of the Articles

The wide-ranging articles in this collection illuminate important features of these intersecting problems. While there is considerable overlap among the articles, there are also important differences, perhaps especially regarding how to differentiate contemporary right-wing populism from left-wing populisms, or indeed whether the term populism should be applied to such left-wing movements at all. Mostov sharply distinguishes these two types of movements, the populist and the popular, while Ackerly draws a distinction between two forms of populism, one, the anti-democratic version, which marginalizes certain groups of “others” and the second, the activist protest movements that mobilize against various forms of structural injustice. These forms of activism can play an important role in countering the anti-democratic movements, even finding some helpful support in feminist philanthropy. Hirschmann agrees that peaceful popular protest is useful in resisting white patriarchal populist authoritarianism. In her analysis, right-wing populism tends to operate outside of government and tends to be directed against it, often with the use of violence, in contrast to left-wing populist movements (including Black Lives Matter), which seek to effect change within government rather than questioning its legitimacy. Yet, while that may be true of many recent left-wing protests, one can ask whether it adequately captures the aims of left-wing anarchist movements or even some contemporary movements aimed at social and economic transformation, understood not only as a prerequisite for fundamental political change but as essential in itself, inasmuch as contemporary social and economic life embodies distinctive forms of structural injustice. Like Hirschmann, Botting is deeply critical of authoritarian populism but retains the term populism for both right-wing and left-wing varieties. In her account, both exemplify similar emphases on “anti-elitism, defense of the common people, and belief in the foundational value of popular sovereignty” (Botting). While this may well be historically accurate, my own inclination would be to leave the term populism to the authoritarian varieties in view of the unclarities in the concept and the prevalence of exclusionary right-wing forms. At least, I would qualify the term populism with the adjective “authoritarian” for these preponderant contemporary cases, a usage I have embraced in this introduction.

The various articles on this research topic cast important light on the gendered nature of contemporary authoritarian populism, although with somewhat different emphases. The gendered and patriarchal nature of populisms and their tendency to oppress not only women but other marginalized groups are stressed in all the articles and are brought to the fore especially in the pieces by Mostov, Ackerly, Hirschmann, and Botting. Populism, on Mostov’s account, is always gendered and dangerous to women and democracy because it creates the nationalist distinction between “us” and “them,” and attempts to force women to reproduce to benefit the “homeland” or the majority race/ethno-nation. It makes extensive use of gendered narratives to perpetuate dominance. Ackerly points to the way anti-democratic populist leaders use patriarchy as a “wellspring for mobilization,” along with other hierarchical ideologies such as racism, caste, and xenophobia, which refocus discontent into resentment of particular groups of people. She especially emphasizes the power of feminist activism and its more inclusive ideologies to counter such populism. Hirschmann calls attention to the violence associated with contemporary patriarchal forms of populism, carried out almost exclusively by white men. Beyond that, she notes, its political violence is driven by white male supremacist ideology, expressing a distorted version of white masculinity, fighting the loss that sexual and racial equality would supposedly bring to their privileges. She argues that protest movements are key to fighting this “deluded populism.” A related diagnosis of male privilege and sense of entitlement is offered by Botting, where the privilege becomes entrenched over time in economic and legal conditions for women’s subordination. She points to the ways in which patriarchal populism exploits stereotypes of masculinity and femininity (and race and class) to demean women and convert them to become servants of men, noting how these tendencies were envisioned as leading to dystopias in the “political science fiction” of Mary Shelley, Octavia Butler, and Margaret Atwood.

The articles by Love and Bracewell draw attention to the women participants in these white right-wing movements, and especially in their extreme white supremacist forms. The authors analyze various ways in which these women lend support to the movements, even if they do not often take leadership roles (with some notable exceptions). Love contends that whether adopting the roles of “Shield Maidens, Fashy Femmes, and TradWives,” white women have been complicit in white supremacy given their continued active participation in these movements. Bracewell focuses on the case of women’s embrace of the QAnon Conspiracy Movement and demonstrates both the role femininity plays in engaging women in populist projects and the importance of developing a more comprehensive understanding of gender and populism. Running through all six articles is a consideration of the intersectional dimensions of these authoritarian populisms and the ways they at one and the same time define the “people” as standing against not only feminists and employed women, but also LGBTQ + groups, migrants, and religious minorities.

A final theme that is highlighted especially in Botting’s discussion of the post-apocalyptic plague literature of Shelly and the other writers concerns the predictive power of their accounts in shedding light on our contemporary pandemic situation. Indeed, the intensification of patriarchal and anti-democratic movements that such pandemics tend to usher in should give us pause as we stand at what seems like a decisive moment in dealing with the COVID-19 plague (Agius et al., 2020). While contemporary science has brought great hope with highly effective vaccines, the political attacks on democracy and the attendant emphasis on disinformation continue unabated. Throughout, we observe how women, with their heavy care responsibilities, are disproportionately impacted, and their rights and capacities to take an equal part in contemporary society have come under grave attack. Yet, at the same time, we see how feminist and anti-racist social movements, led by a new generation committed to establishing genuine equality and deeper forms of democracy, lend substance to the hope that these negative forces can be countered, these structural injustices dismantled, and some measure of real freedom achieved for all.
